# Supplementary material for: The Diagnostic Performance of 2-[18F]FDG PET/CT in Identifying Richter Transformation in Chronic Lymphocytic Leukemia: An Updated Systematic Review and Bivariate Meta-Analysis
Source: Cancers (Basel). 2024 May 5;16(9):1778. doi: 10.3390/cancers16091778 (PMC11083202; doi:10.3390/cancers16091778)
Supplement: Supplementary file 1 [file cancers-16-01778-s001.zip › cancers-2976360-supplementary.pdf]

PRISMA 2020 Checklist

Section and Topic	Item #	Checklist item	Indicate if the item is reported
**TITLE**			
Title	1	Identify the report as a systematic review.	yes
**ABSTRACT**			
Abstract	2	See the PRISMA 2020 for Abstracts checklist.	yes
**INTRODUCTION**			
Rationale	3	Describe the rationale for the review in the context of existing knowledge.	yes
Objectives	4	Provide an explicit statement of the objective(s) or question(s) the review addresses.	yes
**METHODS**			
Eligibility criteria	5	Specify the inclusion and exclusion criteria for the review and how studies were grouped for the syntheses.	yes
Information sources	6	Specify all databases, registers, websites, organisations, reference lists and other sources searched or consulted to identify studies. Specify the date when each source was last searched or consulted.	yes
Search strategy	7	Present the full search strategies for all databases, registers and websites, including any filters and limits used.	yes
Selection process	8	Specify the methods used to decide whether a study met the inclusion criteria of the review, including how many reviewers screened each record and each report retrieved, whether they worked independently, and if applicable, details of automation tools used in the process.	yes
Data collection process	9	Specify the methods used to collect data from reports, including how many reviewers collected data from each report, whether they worked independently, any processes for obtaining or confirming data from study investigators, and if applicable, details of automation tools used in the process.	yes
Data items	10a	List and define all outcomes for which data were sought. Specify whether all results that were compatible with each outcome domain in each study were sought (e.g. for all measures, time points, analyses), and if not, the methods used to decide which results to collect.	yes
	10b	List and define all other variables for which data were sought (e.g. participant and intervention characteristics, funding sources). Describe any assumptions made about any missing or unclear information.	yes
Study risk of bias assessment	11	Specify the methods used to assess risk of bias in the included studies, including details of the tool(s) used, how many reviewers assessed each study and whether they worked independently, and if applicable, details of automation tools used in the process.	yes
Effect measures	12	Specify for each outcome the effect measure(s) (e.g. risk ratio, mean difference) used in the synthesis or presentation of results.	yes
Synthesis methods	13a	Describe the processes used to decide which studies were eligible for each synthesis (e.g. tabulating the study intervention characteristics and comparing against the planned groups for each synthesis (item #5)).	yes
	13b	Describe any methods required to prepare the data for presentation or synthesis, such as handling of missing summary statistics, or data conversions.	yes
	13c	Describe any methods used to tabulate or visually display results of individual studies and syntheses.	yes
	13d	Describe any methods used to synthesize results and provide a rationale for the choice(s). If meta-analysis was performed, describe the model(s), method(s) to identify the presence and extent of statistical heterogeneity, and software package(s) used.	yes
	13e	Describe any methods used to explore possible causes of heterogeneity among study results (e.g. subgroup analysis, meta-regression).	yes
	13f	Describe any sensitivity analyses conducted to assess robustness of the synthesized results.	yes
Reporting bias assessment	14	Describe any methods used to assess risk of bias due to missing results in a synthesis (arising from reporting biases).	yes
Certainty assessment	15	Describe any methods used to assess certainty (or confidence) in the body of evidence for an outcome.	yes

PRISMA 2020 Checklist

Section and Topic	Item #	Checklist item	Indicate if the item is reported
**RESULTS**			
Study selection	16a	Describe the results of the search and selection process, from the number of records identified in the search to the number of studies included in the review, ideally using a flow diagram.	yes
	16b	Cite studies that might appear to meet the inclusion criteria, but which were excluded, and explain why they were excluded.	yes
Study characteristics	17	Cite each included study and present its characteristics.	yes
Risk of bias in studies	18	Present assessments of risk of bias for each included study.	yes
Results of individual studies	19	For all outcomes, present, for each study: (a) summary statistics for each group (where appropriate) and (b) an effect estimate and its precision (e.g. confidence/credible interval), ideally using structured tables or plots.	yes
Results of syntheses	20a	For each synthesis, briefly summarise the characteristics and risk of bias among contributing studies.	yes
	20b	Present results of all statistical syntheses conducted. If meta-analysis was done, present for each the summary estimate and its precision (e.g. confidence/credible interval) and measures of statistical heterogeneity. If comparing groups, describe the direction of the effect.	yes
	20c	Present results of all investigations of possible causes of heterogeneity among study results.	yes
	20d	Present results of all sensitivity analyses conducted to assess the robustness of the synthesized results.	yes
Reporting biases	21	Present assessments of risk of bias due to missing results (arising from reporting biases) for each synthesis assessed.	yes
Certainty of evidence	22	Present assessments of certainty (or confidence) in the body of evidence for each outcome assessed.	yes
**DISCUSSION**			
Discussion	23a	Provide a general interpretation of the results in the context of other evidence.	yes
	23b	Discuss any limitations of the evidence included in the review.	yes
	23c	Discuss any limitations of the review processes used.	yes
	23d	Discuss implications of the results for practice, policy, and future research.	yes
**OTHER INFORMATION**			
Registration and protocol	24a	Provide registration information for the review, including register name and registration number, or state that the review was not registered.	yes
	24b	Indicate where the review protocol can be accessed, or state that a protocol was not prepared.	yes
	24c	Describe and explain any amendments to information provided at registration or in the protocol.	yes
Support	25	Describe sources of financial or non-financial support for the review, and the role of the funders or sponsors in the review.	yes
Competing interests	26	Declare any competing interests of review authors.	yes
Availability of data, code and other materials	27	Report which of the following are publicly available and where they can be found: template data collection forms; data extracted from included studies; data used for all analyses; analytic code; any other materials used in the review.	yes

From: Page MJ, McKenzie JE, Bossuyt PM, Boutron I, Hoffmann TC, Mulrow CD, et al. The PRISMA 2020 statement: an updated guideline for reporting systematic reviews. BMJ 2021;372:n71. doi: 10.1136/bmj.n71

For more information, visit: http://www.prisma-statement.org/

Table S2. list of excluded studies with reasons.

**Excluded Studies**	**Reason for exclusion**
El-Galaly TC, Villa D, Gormsen LC, Baech J, Lo A, Cheah CY. FDG-PET/CT in the management of lymphomas: current status and future directions. J Intern Med. 2018 Oct;284(4):358-376. doi: 10.1111/joim.12813.	Review in the field of interest
Zwanenburg A, Vallières M, Abdalah MA, Aerts HJWL, Andrearczyk V, Apte A, Ashrafinia S, Bakas S, Beukinga RJ, Boellaard R, Bogowicz M, Boldrini L, Buvat I, Cook GJR, Davatzikos C, Depeursinge A, Desseroit MC, Dinapoli N, Dinh CV, Echegaray S, El Naqa I, Fedorov AY, Gatta R, Gillies RJ, Goh V, Götz M, Guckenberger M, Ha SM, Hatt M, Isensee F, Lambin P, Leger S, Leijenaar RTH, Lenkowicz J, Lippert F, Losnegård A, Maier-Hein KH, Morin O, Müller H, Napel S, Nioche C, Orlhac F, Pati S, Pfaehler EAG, Rahmim A, Rao AUK, Scherer J, Siddique MM, Sijtsema NM, Socarras Fernandez J, Spezi E, Steenbakkers RJHM, Tanadini-Lang S, Thorwarth D, Troost EGC, Upadhaya T, Valentini V, van Dijk LV, van Griethuysen J, van Velden FHP, Whybra P, Richter C, Löck S. The Image Biomarker Standardization Initiative: Standardized Quantitative Radiomics for High-Throughput Image-based Phenotyping. Radiology. 2020 May;295(2):328-338. doi: 10.1148/radiol.2020191145.	Original study not in the field of interest
Varasteh Z, Mohanta S, Robu S, Braeuer M, Li Y, Omidvari N, Topping G, Sun T, Nekolla SG, Richter A, Weber C, Habenicht A, Haberkorn UA, Weber WA. Molecular Imaging of Fibroblast Activity After Myocardial Infarction Using a ⁶⁸Ga-Labeled Fibroblast Activation Protein Inhibitor, FAPI-04. J Nucl Med. 2019 Dec;60(12):1743-1749. doi: 10.2967/jnumed.119.226993.	Original study not in the field of interest
Liu Y. Unusual Illustration of Richter Transformation in Chronic Lymphocytic Leukemia on FDG PET/CT. Clin Nucl Med. 2022 Aug 1;47(8):746-747. doi: 10.1097/RLU.0000000000004125.	Case report in the field of interest
Rossi D, Gaidano G. Richter syndrome. Adv Exp Med Biol. 2013;792:173-91. doi:10.1007/978-1-4614-8051-8_8.	Review in the field of interest
Barré L, Hovhannisyan N, Bodet-Milin C, Kraeber-Bodéré F, Damaj G. [¹⁸F]-Fludarabine for Hematological Malignancies. Front Med (Lausanne). 2019 Apr 17;6:77. doi: 10.3389/fmed.2019.00077.	Review not in the field of interest
Condoluci A, Rossi D. Treatment of Richter's Syndrome. Curr Treat Options Oncol. 2017 Nov 21;18(12):75. doi: 10.1007/s11864-017-0512-y.	Review not in the field of interest
Shaikh F, Janjua A, Van Gestel F, Ahmad A. Richter Transformation of Chronic Lymphocytic Leukemia: A Review of Fluorodeoxyglucose Positron Emission Tomography-Computed Tomography and Molecular Diagnostics. Cureus. 2017 Jan 9;9(1):e968. doi: 10.7759/cureus.968.	Review in the field of interest
Khan M, Siddiqi R, Thompson PA. Approach to Richter	Review not in the field of interest

transformation of chronic lymphocytic leukemia in the era of novel therapies. Ann Hematol. 2018 Jan;97(1):1-15. doi: 10.1007/s00277-017-3149-9	
Zhao Z, Hu Y, Li J, Zhou Y, Zhang B, Deng S. Applications of PET in Diagnosis and Prognosis of Leukemia. Technol Cancer Res Treat. 2020 Jan-Dec;19:1533033820956993. doi: 10.1177/1533033820956993.	Review in the field of interest
Rossi D, Gaidano G. Richter syndrome: pathogenesis and management. Semin Oncol. 2016 Apr;43(2):311-9. doi: 10.1053/j.seminoncol.2016.02.012.	Review in the field of interest
Chen W, Jeudy J. Assessment of Myocarditis: Cardiac MR, PET/CT, or PET/MR? Curr Cardiol Rep. 2019 Jun 26;21(8):76. doi: 10.1007/s11886-019-1158-0.	Review not in the field of interest
Zirakchian Zadeh M. Clinical Application of ¹⁸F-FDG-PET Quantification in Hematological Malignancies: Emphasizing Multiple Myeloma, Lymphoma and Chronic Lymphocytic Leukemia. Clin Lymphoma Myeloma Leuk. 2023 Nov;23(11):800-814. doi: 10.1016/j.clml.2023.07.008.	Review not in the field of interest
Rossi D. Richter's syndrome: Novel and promising therapeutic alternatives. Best Pract Res Clin Haematol. 2016 Mar;29(1):30-39. doi:10.1016/j.beha.2016.08.006.	Review not in the field of interest
Sood A, Parihar AS, Lad D, Kumar R, Singh H, Mittal BR. An Unusual Presentation of Richter's Transformation of Chronic Lymphocytic Leukemia in Liver and Lung on ¹⁸F-Labeled Fluoro-2-Deoxyglucose Positron Emission Tomography/Computed Tomography. Indian J Nucl Med. 2020 Jan-Mar;35(1):70-71. doi: 10.4103/ijnm.IJNM_175_19.	Case report in the field of interest
Albano D, Dondi F, Bertagna F, Treglia G. The Role of [⁶⁸Ga]Ga-Pentixafor PET/CT or PET/MRI in Lymphoma: A Systematic Review. Cancers (Basel). 2022 Aug 5;14(15):3814. doi: 10.3390/cancers14153814.	Review not in the field of interest
Molica S. FDG/PET in CLL today. Blood. 2014 May 1;123(18):2749-50. doi: 10.1182/blood-2014-03-563700.	Editorial in the field of interest
Federmann B, Mueller MR, Steinhilber J, Horger MS, Fend F. Diagnosis of Richter transformation in chronic lymphocytic leukemia: histology tips the scales. Ann Hematol. 2018 Oct;97(10):1859-1868. doi: 10.1007/s00277-018-3390-x.	Original article not in the field of interest
Sanz G, Rioja J, Zudaire JJ, Berián JM, Richter JA. PET and prostate cancer. World J Urol. 2004 Nov;22(5):351-2. doi: 10.1007/s00345-004-0418-8.	Review not in the field of interest
Hirshoren N, Olayos E, Herschtal A, Ravi Kumar AS, Gyorki DE. Preoperative Positron Emission Tomography for Node-Positive Head and Neck Cutaneous Squamous Cell Carcinoma. Otolaryngol Head Neck Surg. 2018 Jan;158(1):122-126. doi: 10.1177/0194599817731735.	Original article not in the field of interest
Yuen R, Wagner M, Richter S, Dufour J, Wuest M, West FG,	Original article not in the field of interest

Wuest F. Design, synthesis, and evaluation of positron emission tomography/fluorescence dual imaging probes for targeting facilitated glucose transporter 1 (GLUT1). Org Biomol Chem. 2021 Apr 14;19(14):3241-3254. doi: 10.1039/d1ob00199j.	
Dixon JG, Dimier N, Nielsen T, Zheng J, Marcus R, Morschhauser F, Evens AM, Federico M, Blum KA, Shi Q. End of induction positron emission tomography complete response (PET-CR) as a surrogate for progression-free survival in previously untreated follicular lymphoma. Br J Haematol. 2022 Jul;198(2):333-337. doi: 10.1111/bjh.18217.	Original article not in the field of interest
Gupta N, Mittal A, Duggal R, Dadu T, Agarwal A, Handoo A. Hodgkin Variant of Richter's Transformation in Chronic Lymphocytic Leukemia (CLL): An Illustrative Case Report and Literature Review. Int J Hematol Oncol Stem Cell Res. 2021 Oct 1;15(4):249-254. doi: 10.18502/ijhoscr.v15i4.7480.	Original article not in the field of interest
Johansson P, Alig S, Richter J, Hanoun C, Rekowski J, Dürig J, Ylstra B, de Jong D, Klapper W, Alizadeh AA, Dührsen U, Hüttmann A. Outcome prediction by interim positron emission tomography and IgM monoclonal gammopathy in diffuse large B-cell lymphoma. Ann Hematol. 2023 Dec;102(12):3445-3455. doi: 10.1007/s00277-023-05393-1.	Original article not in the field of interest
Fjordside L, Mens H, Asmar A. Using 18F-FDG PET/CT to rule out Richter transformation as cause of deterioration in a patient with chronic lymphatic leukemia and severe COVID-19: A case report. Medicine (Baltimore). 2021 Nov 5;100(44):e27545. doi: 10.1097/MD.0000000000027545.	Case report in the field of interest
Basu S, Alavi A. PET-Based Personalized Management in Clinical Oncology: An Unavoidable Path for the Foreseeable Future. PET Clin. 2016 Jul;11(3):203-7. doi: 10.1016/j.cpet.2016.03.002.	Review not in the field of interest
Alessandrino F, DiPiro PJ, Jagannathan JP, Babina G, Krajewski KM, Ramaiya NH, Giardino AA. Multimodality imaging of indolent B cell lymphoma from diagnosis to transformation: what every radiologist should know. Insights Imaging. 2019 Feb 22;10(1):25. doi: 10.1186/s13244-019-0705-y.	Review not in the field of interest
London J, Grados A, Fermé C, Charmillon A, Maurier F, Deau B, Crickx E, Brice P, Chapelon-Abric C, Haioun C, Burroni B, Alifano M, Le Jeunne C, Guillevin L, Costedoat-Chalumeau N, Schleinitz N, Mouthon L, Terrier B. Sarcoidosis occurring after lymphoma: report of 14 patients and review of the literature. Medicine (Baltimore). 2014 Nov;93(21):e121. doi: 10.1097/MD.0000000000000121.	Case report not in the field of interest
Swords R, Bruzzi J, Giles F. Recent advances in the diagnosis and therapy of Richter's syndrome. Med Oncol. 2007;24(1):17-32. doi: 10.1007/BF02685899.	Review not in the field of interest
Chantepie S, Hovhannisyan N, Guillouet S, Pelage JP, Ibazizene M, Bodet-Milin C, Carlier T, Gac AC, Réboursière E, Vilque JP,	Original article not in the field of interest

Kraeber-Bodéré F, Manrique A, Damaj G, Leporrier M, Barré L. ¹⁸F-Fludarabine PET for Lymphoma Imaging: First-in-Humans Study on DLBCL and CLL Patients. J Nucl Med. 2018 Sep;59(9):1380-1385. doi: 10.2967/jnumed.117.206920.	
Broecker-Preuss M, Becher-Boveleth N, Müller SP, Hüttmann A, Hanoun C, Grafe H, Richter J, Klapper W, Rekowski J, Bockisch A, Dührsen U. Impact of germline polymorphisms in genes regulating glucose uptake on positron emission tomography findings and outcome in diffuse large B-cell lymphoma: results from the PETAL trial. J Cancer Res Clin Oncol. 2022 Oct;148(10):2611-2621. doi:10.1007/s00432-021-03796-z.	Original article not in the field of interest
Giannoni P, Marini C, Cutrona G, Todoerti K, Neri A, Ibatici A, Sambuceti G, Pigozzi S, Mora M, Ferrarini M, Fais F, de Totero D. A High Percentage of CD16+ Monocytes Correlates with the Extent of Bone Erosion in Chronic Lymphocytic Leukemia Patients: The Impact of Leukemic B Cells in Monocyte Differentiation and Osteoclast Maturation. Cancers (Basel). 2022 Dec 3;14(23):5979. doi:10.3390/cancers14235979.	Original article not in the field of interest
Ayubcha C, Hosoya H, Seraj SM, Zadeh MZ, Werner T, Alavi A. The role of ¹⁸F-FDG PET in the assessment of a benign hematological disorder: polycythemia. Hell J Nucl Med. 2019 Jan-Apr;22(1):4-5. doi:10.1967/s002449910951	Editorial not in the field of interest
Albano D, Ferro P, Bosio G, Fallanca F, Re A, Tucci A, Maria Ferreri AJ, Angelillo P, Gianolli L, Giubbini R, Bertagna F. Diagnostic and Clinical Impact of Staging ¹⁸F-FDG PET/CT in Mantle-Cell Lymphoma: A Two-Center Experience. Clin Lymphoma Myeloma Leuk. 2019 Aug;19(8):e457-e464. doi: 10.1016/j.clml.2019.04.016.	Original article not in the field of interest
MacManus MP, Hofman MS, Hicks RJ, Campbell BA, Wirth A, Seymour JF, Haynes N, Burbury K. Abscopal Regressions of Lymphoma After Involved-Site Radiation Therapy Confirmed by Positron Emission Tomography. Int J Radiat Oncol Biol Phys. 2020 Sep 1;108(1):204-211. doi: 10.1016/j.ijrobp.2020.02.636.	Original article not in the field of interest
Prexler C, Knape MS, Erlewein-Schweizer J, Roll W, Specht K, Woertler K, Weichert W, von Luettichau I, Rossig C, Hauer J, Richter GHS, Weber W, Burdach S. Correlation of Transcriptomics and FDG-PET SUVmax Indicates Reciprocal Expression of Stemness-Related Transcription Factor and Neuropeptide Signaling Pathways in Glucose Metabolism of Ewing Sarcoma. Cancers (Basel). 2022 Dec 5;14(23):5999. doi: 10.3390/cancers14235999.	Original article not in the field of interest
Sanz G, Robles JE, Giménez M, Arocena J, Sánchez D, Rodriguez-Rubio F, Rosell D, Richter JA, Berián JM. Positron emission tomography with 18fluorine-labelled deoxyglucose: utility in localized and advanced prostate cancer. BJU Int. 1999 Dec;84(9):1028-31. doi: 10.1046/j.1464-410x.1999.00349.x.	Original article not in the field of interest
Gajendra S, Sharma R, Sahoo MK. Triple cancer: chronic	Case report not in the field of interest

lymphocytic leukemia with bladder and prostate carcinoma. Malays J Pathol. 2015 Aug;37(2):159-63.	
Yılmaz S, Ozhan M, Asa S, Sağer MS, Biricik FS, Halaç M, Sönmezoğlu K. Detection of Hodgkin Transformation in a Case of Chronic Lymphocytic Leukemia by PET/CT. Mol Imaging Radionucl Ther. 2014 Jun;23(2):67-9. doi: 10.4274/mirt.362.	Case report in the field of interest
Rezvani S, Tominna M, Al-Katib S, Smith MD, Cousineau C, Al-Katib A. Lymphomatoid Granulomatosis in a Patient with Chronic Lymphocytic Leukemia and Rapidly Progressing Peribronchovascular Pulmonary Infiltrates. Case Rep Pulmonol. 2019 Jan 21;2019:9870494. doi: 10.1155/2019/9870494	Case report in the field of interest
Martí-Climent JM, Prieto E, Morán V, Sancho L, Rodríguez-Fraile M, Arbizu J, García-Velloso MJ, Richter JA. Effective dose estimation for oncological and neurological PET/CT procedures. EJNMMI Res. 2017 Dec;7(1):37. doi: 10.1186/s13550-017-0272-5	Original article not in the field of interest
Lavalle M, Minordi LM, Hohaus S, Manfredi R, Rufini V. Richter Syndrome Presenting With Colon Localization. Clin Nucl Med. 2019 Feb;44(2):e87-e89. doi: 10.1097/RLU.0000000000002393.	Case report in the field of interest
Fiz F, Marini C, Piva R, Miglino M, Massollo M, Bongioanni F, Morbelli S, Bottoni G, Campi C, Bacigalupo A, Bruzzi P, Frassoni F, Piana M, Sambuceti G. Adult advanced chronic lymphocytic leukemia: computational analysis of whole- body CT documents a bone structure alteration. Radiology. 2014 Jun;271(3):805-13. doi: 10.1148/radiol.14131944.	Original article not in the field of interest
Bastarrika G, García-Velloso MJ, Lozano MD, Montes U, Torre W, Spiteri N, Campo A, Seijo L, Alcaide AB, Pueyo J, Cano D, Vivas I, Cosín O, Domínguez P, Serra P, Richter JA, Montuenga L, Zulueta JJ. Early lung cancer detection using spiral computed tomography and positron emission tomography. Am J Respir Crit Care Med. 2005 Jun 15;171(12):1378-83. doi: 10.1164/rccm.200411-1479OC	Original article not in the field of interest
Richter N, Nellessen N, Dronse J, Dillen K, Jacobs HIL, Langen KJ, Dietlein M, Kracht L, Neumaier B, Fink GR, Kukolja J, Onur OA. Spatial distributions of cholinergic impairment and neuronal hypometabolism differ in MCI due to AD. Neuroimage Clin. 2019;24:101978. doi: 10.1016/j.nicl.2019.101978.	Original article not in the field of interest
Hodgson YA, Jones SG, Knight H, Sovani V, Fox CP. Herpes Simplex Necrotic Lymphadenitis Masquerading as Richter's Transformation in Treatment-Naïve Patients With Chronic Lymphocytic Leukemia. J Hematol. 2019 Jun;8(2):79-82. doi: 10.14740/jh517.	Original article not in the field of interest
Casquero-Veiga M, Romero-Miguel D, MacDowell KS, Torres-Sanchez S, Garcia-Partida JA, Lamanna-Rama N, Gómez-Rangel V, Romero-Miranda A, Berrocoso E, Leza JC, Arango C, Desco M, Soto-Montenegro ML. Omega-3 fatty acids during adolescence prevent schizophrenia-related behavioural deficits:	Original article not in the field of interest

Neurophysiological evidences from the prenatal viral infection with PolyI:C. Eur Neuropsychopharmacol. 2021 May;46:14-27. doi: 10.1016/j.euroneuro.2021.02.001.	
Richter JA, Rodríguez M, Rioja J, Peñuelas I, Martí-Climent J, Garrastachu P, Quincoces G, Zudaire J, García-Velloso MJ. Dual tracer 11C-choline and FDG-PET in the diagnosis of biochemical prostate cancer relapse after radical treatment. Mol Imaging Biol. 2010 Apr;12(2):210-7. doi: 10.1007/s11307-009-0243-y.	Original article not in the field of interest
Prieto E, García-Velloso MJ, Rodríguez-Fraile M, Morán V, García-García B, Guillén F, Morales MI, Sancho L, Peñuelas I, Richter JÁ, Martí-Climent JM. Significant dose reduction is feasible in FDG PET/CT protocols without compromising diagnostic quality. Phys Med. 2018 Feb;46:134-139. doi: 10.1016/j.ejmp.2018.01.021.	Original article not in the field of interest
Alfonso A, Redondo M, Rubio T, Del Olmo B, Rodríguez-Wilhelmi P, García-Velloso MJ, Richter JA, Páramo JA, Lecumberri R. Screening for occult malignancy with FDG-PET/CT in patients with unprovoked venous thromboembolism. Int J Cancer. 2013 Nov;133(9):2157-64. doi: 10.1002/ijc.28229.	Original article not in the field of interest
Ulaner GA, Sawan P. Transient Osteoporosis of the Hip on FDG PET/CT. Clin Nucl Med. 2017 May;42(5):401-402. doi: 10.1097/RLU.0000000000001630.	Case report not in the field of interest
Pudis M, Gràcia-Sánchez L, Muñoz-Palomar A, Sánchez-Rodríguez IE, Cortés-Romera M. Richter's transformation with isolated cardiac involvement diagnosed by 2-[¹⁸F]FDG PET/CT scan. J Nucl Cardiol. 2023 Feb;30(1):427-430. doi: 10.1007/s12350-021-02845-x.	Case report in the field of interest
García Vicente AM, Jiménez Londoño GA, Hernández Ruiz B, Soriano Castrejón Á. Richter's syndrome: Utility of ^{18}F-FDG PET/CT. Rev Esp Med Nucl Imagen Mol. 2013 Mar;32(2):102-4. doi: 10.1016/j.remn.2012.08.001.	Case report in the field of interest
Marini C, Bruno S, Fiz F, Campi C, Piva R, Cutrona G, Matis S, Nieri A, Miglino M, Ibatici A, Maria Orengo A, Maria Massone A, Neumaier CE, Totero D, Giannoni P, Bauckneht M, Pennone M, Tenca C, Gugiatti E, Bellini A, Borra A, Tedone E, Efetürk H, Rosa F, Emionite L, Cilli M, Bagnara D, Brucato V, Bruzzi P, Piana M, Fais F, Sambuceti G. Functional Activation of Osteoclast Commitment in Chronic Lymphocytic Leukaemia: a Possible Role for RANK/RANKL Pathway. Sci Rep. 2017 Oct 26;7(1):14159. doi: 10.1038/s41598-017-12761-1.	Original article not in the field of interest
Okada J, Yoshikawa K, Imazeki K, Minoshima S, Uno K, Itami J, Kuyama J, Maruno H, Arimizu N. The use of FDG-PET in the detection and management of malignant lymphoma: correlation of uptake with prognosis. J Nucl Med. 1991 Apr;32(4):686-91.	Original article not in the field of interest
Zschaeck S, Löck S, Leger S, Haase R, Bandurska-Luque A, Appold S, Kotzerke J, Zips D, Richter C, Gudziol V, Schreiber A,	Original article not in the field of interest

Zöphel K, Baumann M, Krause M. FDG uptake in normal tissues assessed by PET during treatment has prognostic value for treatment results in head and neck squamous cell carcinomas undergoing radiochemotherapy. Radiother Oncol. 2017 Mar;122(3):437-444. doi: 10.1016/j.radonc.2017.01.019.	
Yamane T, Park MJ, Richter D, Nekolla SG, Javadi MS, Lapa C, Samnick S, Buck AK, Herrmann K, Higuchi T. Small-animal PET imaging of isolated perfused rat heart. J Nucl Med. 2014 Mar;55(3):495-9. doi: 10.2967/jnumed.113.129429.	Original article not in the field of interest
Merdin A, Yıldız J, Dal Mehmet S, Çakar MK, Batgi H, Tekgündüz E, Onursever A, Altuntaş F. A 80-Year-Old Woman with B-Cell Prolymphocytic Leukemia. Hematol Rep. 2017 Mar 22;9(1):6995. doi: 10.4081/hr.2017.6995.	Case report not in the field of interest
Arbizu J, Prieto E, Martínez-Lage P, Martí-Climent JM, García-Granero M, Lamet I, Pastor P, Riverol M, Gómez-Isla MT, Peñuelas I, Richter JA, Weiner MW; Alzheimer's Disease Neuroimaging Initiative. Automated analysis of FDG PET as a tool for single-subject probabilistic prediction and detection of Alzheimer's disease dementia. Eur J Nucl Med Mol Imaging. 2013 Sep;40(9):1394-405. doi:10.1007/s00259-013-2458-z.	Original article not in the field of interest
Kader I, Leavers B, Shashinder S, Wylie B, Chi KK, Sundaresan P. Synchronous or metachronous lymphoma and metastatic cutaneous squamous cell carcinoma in the head and neck region: a diagnostic and management dilemma. J Laryngol Otol. 2016 Jul;130 Suppl 4:S45-9. doi: 10.1017/S002221511600832X.	Original article not in the field of interest
Panizo C, Pérez-Salazar M, Bendandi M, Rodríguez-Calvillo M, Boán JF, García-Velloso MJ, Richter J, Rocha E. Positron emission tomography using 18F-fluorodeoxyglucose for the evaluation of residual Hodgkin's disease mediastinal masses. Leuk Lymphoma. 2004 Sep;45(9):1829-33. doi: 10.1080/10428190420002223813.	Original article not in the field of interest
Prieto E, Martí-Climent JM, Morán V, Sancho L, Barbés B, Arbizu J, Richter JA. Brain PET imaging optimization with time of flight and point spread function modelling. Phys Med. 2015 Dec;31(8):948-955. doi: 10.1016/j.ejmp.2015.07.001.	Original article not in the field of interest
Tiling R, Linke R, Untch M, Richter A, Fieber S, Brinkbäumer K, Tatsch K, Hahn K. 18F-FDG PET and 99mTc-sestamibi scintimammography for monitoring breast cancer response to neoadjuvant chemotherapy: a comparative study. Eur J Nucl Med. 2001 Jun;28(6):711-20. doi: 10.1007/s002590100539.	Original article not in the field of interest
Krarup MMK, Nygård L, Vogelius IR, Andersen FL, Cook G, Goh V, Fischer BM. Heterogeneity in tumours: Validating the use of radiomic features on ¹⁸F-FDG PET/CT scans of lung cancer patients as a prognostic tool. Radiother Oncol. 2020 Mar;144:72-78. doi: 10.1016/j.radonc.2019.10.012.	Original article not in the field of interest
Abdulqadhr G, Molin D, Aström G, Suurküla M, Johansson L,	Original article not in the field of interest

Hagberg H, Ahlström H. Whole-body diffusion-weighted imaging compared with FDG-PET/CT in staging of lymphoma patients. Acta Radiol. 2011 Mar 1;52(2):173-80. doi: 10.1258/ar.2010.100246.	
Bandurska-Luque A, Löck S, Haase R, Richter C, Zöphel K, Perrin R, Appold S, Krause M, Steinbach J, Kotzerke J, Hofheinz F, Zips D, Baumann M, Troost EGC. Correlation between FMISO-PET based hypoxia in the primary tumour and in lymph node metastases in locally advanced HNSCC patients. Clin Transl Radiat Oncol. 2019 Feb 15;15:108-112. doi: 10.1016/j.ctro.2019.02.002.	Original article not in the field of interest
Jacobs SA, Harrison AM, Swerdlow SH, Foon KA, Avril N, Vidnovic N, Joyce J, DeMonaco N, McCarty KS Jr. Radioisotopic localization of (90)Yttrium-ibritumomab tiuxetan in patients with CD20+ non-Hodgkin's lymphoma. Mol Imaging Biol. 2009 Jan-Feb;11(1):39-45. doi: 10.1007/s11307-008-0170-3.	Original article not in the field of interest
García-Velloso MJ, López G, Galán MJ, Meiriño R, Martí JM, Boán J, Richter JA. Valor clínico de la tomografía de emisión de positrones con F-18-FDG en el seguimiento de pacientes con cáncer de ovario [Clinical value of positron emission tomography with F-18-FDG in the follow up of patients with cancer of the ovary]. An Sist Sanit Navar. 2002 Jan-Apr;25(1):21-9. Spanish. doi: 10.23938/ASSN.0788.	Original article not in the field of interest
Ozturk K, Ustun C, Khaja S, Li F, Rischall M, Cayci Z. Concomitant cutaneous squamous cell carcinoma and chronic lymphocytic leukemia in a patient: The utility of ¹⁸ F-FDG PET/CT in differentiation of nodal metastasis. Am J Hematol. 2018 Aug;93(4):597-598. doi: 10.1002/ajh.25041.	Case report not in the field of interest
Israel I, Richter D, Stritzker J, van Ooschot M, Donat U, Buck AK, Samnick S. PET imaging with [^{68}Ga]NOTA-RGD for prostate cancer: a comparative study with [^{18}F]fluorodeoxyglucose and [^{18}F]fluoroethylcholine. Curr Cancer Drug Targets. 2014;14(4):371-9. doi: 10.2174/1568009614666140403123452.	Original article not in the field of interest
Eichler M, Richter S, Hohenberger P, Kasper B, Andreou D, Heidt V, Bornhäuser M, Schmitt J, Schuler MK. Current State of Sarcoma Care in Germany: Results of an Online Survey of Physicians. Oncol Res Treat. 2019;42(11):589-598. doi: 10.1159/000502758.	Original article not in the field of interest
Richter S, Wuest M, Bergman CN, Way JD, Krieger S, Rogers BE, Wuest F. Rerouting the metabolic pathway of (18)F-labeled peptides: the influence of prosthetic groups. Bioconjug Chem. 2015 Feb 18;26(2):201-12. doi: 10.1021/bc500599m.	Original article not in the field of interest
Tsai SY, Shiau YC, Wang SY, Wu YW. Conjunctival Melanoma on 18F-FDG PET/CT as a Second Primary Cancer. Clin Nucl Med. 2016 Mar;41(3):237-8. doi: 10.1097/RLU.0000000000001082.	Case report not in the field of interest
Calvo R, Martí-Climent JM, Richter JA, Peñuelas I, Crespo-Jara A, Villar LM, García-Velloso MJ. Three-dimensional clinical PET in	Original article not in the field of interest

lung cancer: validation and practical strategies. J Nucl Med. 2000 Mar;41(3):439-48.	
Salem U, Zhang L, Jorgensen JL, Kumar R, Amini B. Adhesive capsulitis mimicking metastasis on 18F-FDG-PET/CT. Clin Nucl Med. 2015 Feb;40(2):e145-7. doi: 10.1097/RLU.0000000000000524	Case report not in the field of interest
Garcia-Velloso MJ, Bastarrika G, de-Torres JP, Lozano MD, Sanchez-Salcedo P, Sancho L, Nuñez-Cordoba JM, Campo A, Alcaide AB, Torre W, Richter JA, Zulueta JJ. Assessment of indeterminate pulmonary nodules detected in lung cancer screening: Diagnostic accuracy of FDG PET/CT. Lung Cancer. 2016 Jul;97:81-6. doi: 10.1016/j.lungcan.2016.04.025.	Original article not in the field of interest
Douglas AP, Atarod M, Prince HM. FDG-PET/CT findings, the vital clue to rare diagnosis of herpes simplex virus lymphadenitis simulating Richter transformation. Pathology. 2019 Jan;51(1):102-104. doi: 10.1016/j.pathol.2018.08.016.	Case report in the field of interest
Glazebrook KN, Zingula S, Jones KN, Fazzio RT. Breast imaging findings in haematological malignancies. Insights Imaging. 2014 Dec;5(6):715-22. doi: 10.1007/s13244-014-0344-2.	Original article not in the field of interest
Roolf C, Richter A, Konkolefski C, Knuebel G, Sekora A, Krohn S, Stenzel J, Krause BJ, Vollmar B, Murua Escobar H, Junghanss C. Decitabine demonstrates antileukemic activity in B cell precursor acute lymphoblastic leukemia with MLL rearrangements. J Hematol Oncol. 2018 May 4;11(1):62. doi: 10.1186/s13045-018-0607-3.	Original article not in the field of interest
Chen Q, Zhang J, Huang H, Qiu T, Jin Z, Shi Y, Zhu H, Fan L, Li J, Shi W, Miao Y. Histiocytic necrotizing lymphadenitis with hemophagocytic lymphohistiocytosis in adults: A single-center analysis of 5 cases. Immun Inflamm Dis. 2024 Feb;12(2):e1202. doi: 10.1002/iid3.1202.	Case series not in the field of interest
Reddy CG, Mauermann ML, Solomon BM, Ringler MD, Jerath NU, Begna KH, Amrami KK, Spinner RJ. Neuroleukemiosis: an unusual cause of peripheral neuropathy. Leuk Lymphoma. 2012 Dec;53(12):2405-11. doi: 10.3109/10428194.2012.691480.	Case report not in the field of interest
Bleeker-Rovers CP, Vos FJ, de Kleijn EMHA, Mudde AH, Dofferhoff TSM, Richter C, Smilde TJ, Krabbe PFM, Oyen WJG, van der Meer JWM. A prospective multicenter study on fever of unknown origin: the yield of a structured diagnostic protocol. Medicine (Baltimore). 2007 Jan;86(1):26-38. doi: 10.1097/MD.0b013e31802fe858.	Original article not in the field of interest
Richter JA, García-Velloso MJ, Domínguez I, Quincoces G, Prieto E, Rodríguez Fraile M. Tomografía por emisión de positrones en el cáncer de mama [Positron emission tomography in breast cancer]. Rev Med Univ Navarra. 2008 Jan-Mar;52(1):4-12.	Review not in the field of interest
Richter JP, Goroncy AK, Ronimus RS, Sutherland-Smith AJ. The Structural and Functional Characterization of Mammalian ADP-dependent Glucokinase. J Biol Chem. 2016 Feb 19;291(8):3694-704. doi: 10.1074/jbc.M115.679902.	Original article not in the field of interest

Prieto E, Domínguez-Prado I, García-Velloso MJ, Peñuelas I, Richter JÁ, Martí-Climent JM. Impact of time-of-flight and point-spread-function in SUV quantification for oncological PET. Clin Nucl Med. 2013 Feb;38(2):103-9. doi: 10.1097/RLU.0b013e318279b9df.	Original article not in the field of interest
Kritikos N, Priftakis D, Stavrinides S, Kleanthous S, Sarafianou E. Nuclear medicine techniques in Merkel cell carcinoma: A case report and review of the literature. Oncol Lett. 2015 Sep;10(3):1610-1616. doi: 10.3892/ol.2015.3377.	Case report not in the field of interest
García Velloso MJ, Quesada J, Martí JM, Azinovic I, Peñuelas I, Alcalde J, Richter JA. Tomografía de emisión de positrones con F-18-FDG: una nueva técnica en la evaluación de pacientes con neoplasias de cabeza y cuello [Positron emission tomography with F-18-FDG: a new tool in the evaluation of patients with head and neck tumors]. An Sist Sanit Navar. 1999 May-Aug;22(2):155-65.	Original article not in the field of interest
Iğdem S, Okkan S, Unalan B, Iğdem A, Ferhanoğlu B. Cervical cancer coexisting with small lymphocytic lymphoma detected during positron emission tomography/computed tomography simulation: a case report. Eur J Gynaecol Oncol. 2008;29(4):405-7.	Case report not in the field of interest
Prieto E, Martí-Climent JM, Gómez-Fernández M, García-Velloso MJ, Valero M, Garrastachu P, Aristu J, Alcázar JL, Torre W, Hernández JL, Pardo FJ, Peñuelas I, Richter JA. Validación de técnicas de segmentación para la tomografía por emisión de positrones mediante imágenes ex-vivo de piezas quirúrgicas oncológicas [Validation of segmentation techniques for positron emission tomography using ex-vivo images of oncological surgical specimens]. Rev Esp Med Nucl Imagen Mol. 2014 Mar-Apr;33(2):79-86. Spanish. doi: 10.1016/j.remn.2013.06.010.	Original article not in the field of interest
Li Y, Wei J, Mao X, Gao Q, Liu L, Cheng P, Liu L, Zhang X, Zhang K, Wang J, Zhu L, Zhou J, Zhang Y, Meng L, Sun H, Li D, Huang M, Huang W, Deng J, Zhang D. Flow Cytometric Immunophenotyping Is Sensitive for the Early Diagnosis of De Novo Aggressive Natural Killer Cell Leukemia (ANKL): A Multicenter Retrospective Analysis. PLoS One. 2016 Aug 2;11(8):e0158827. doi: 10.1371/journal.pone.0158827.	Original article not in the field of interest
Osman MM, Altinyay ME, Abdelmalik AG, Brickman TM, Varvares MA, Nguyen NC. FDG PET/CT incidental diagnosis of a synchronous bladder cancer as a fourth malignancy in a patient with head and neck cancer. Clin Nucl Med. 2011 Jun;36(6):496-7. doi: 10.1097/RLU.0b013e318217393f.	Case report not in the field of interest
Thom AR, Hamerschlak N, Teles VG, Osawa A, Santos FP, Pasqualin Dda C, Wagner J, Yamaga LY, Cunha ML, Campos Neto Gde C, Funari MB. Normalization of lymphocyte count after high ablative dose of I-131 in a patient with chronic lymphoid leukemia and secondary papillary carcinoma of the	Case report not in the field of interest

thyroid. Case report. Einstein (Sao Paulo). 2014 Jan-Mar;12(1):100-5. doi: 10.1590/s1679-45082014rc2657.	
Dillen KNH, Jacobs HIL, Kukolja J, Richter N, von Reutern B, Onur ÖA, Langen KJ, Fink GR. Functional Disintegration of the Default Mode Network in Prodromal Alzheimer's Disease. J Alzheimers Dis. 2017;59(1):169-187. doi: 10.3233/JAD-161120.	Original article not in the field of interest
Richter JA, García-Velloso MJ, Pérez-Equiza C, Gámez C, Pérez-Calvo J, Gorosquieta A, Crespo A. PET-FDG Versus CT in the Staging and Detection of Relapse in Patients with Hodgkin's Disease or Non-Hodgkin Lymphoma. Two Years Experience. Clin Positron Imaging. 1998 Sep;1(4):248. doi: 10.1016/s1095-0397(98)00038-7.	Original article not in the field of interest
Rasmussen JH, Håkansson K, Rasmussen GB, Vogelius IR, Friborg J, Fischer BM, Bentzen SM, Specht L. A clinical prognostic model compared to the newly adopted UICC staging in an independent validation cohort of P16 negative/positive head and neck cancer patients. Oral Oncol. 2018 Jun;81:52-60. doi: 10.1016/j.oraloncology.2018.04.009.	Original article not in the field of interest
Kiyosawa M, Bosley TM, Alavi A, Gupta N, Rhodes CH, Chawluk J, Kushner M, Savino PJ, Sergott RC, Schatz NJ, et al. Positron emission tomography in a patient with progressive multifocal leukoencephalopathy. Neurology. 1988 Dec;38(12):1864-7. doi: 10.1212/wnl.38.12.1864.	Case report not in the field of interest
Richter JA, Torre W, Gámez C, Aramendia JM, Crespo A, Nicolás A, Brugarolas A. Valor de la tomografía por emisión de positrones (PET)-18FDG en el cáncer de pulmón [Value of Pet-18FDG in lung cancer]. Med Clin (Barc). 1999 Nov 6;113(15):567-71.	Original article not in the field of interest
Maffione AM, Rampin L, Paolini R, Rodella E, Lisato LC, Ballotta MR, Pavanato G, Montesi G, Colletti PM, Rubello D. Epstein-Barr Virus-Positive Mucocutaneous Ulcer Mimicking Rectal Carcinoma at 18F-FDG PET/CT. Clin Nucl Med. 2017 Aug;42(8):645-646. doi: 10.1097/RLU.0000000000001725.	Case report not in the field of interest
García-Velloso MJ, Jurado M, Ceamanos C, Aramendía JM, Garrastachu MP, López-García G, Richter JA. Diagnostic accuracy of FDG PET in the follow-up of platinum-sensitive epithelial ovarian carcinoma. Eur J Nucl Med Mol Imaging. 2007 Sep;34(9):1396-405. doi: 10.1007/s00259-007-0366-9.	Original article not in the field of interest

Dyer MJ, Majid A, Walewska R, Gesk S, Harder L, Siebert R, Morgan B. Splenic infarction associated with rapidly progressive chronic lymphocytic leukemia with complex karyotype and ATM mutation. Leuk Res. 2011 May;35(5):e55-7. doi: 10.1016/j.leukres.2010.10.033.	Case report not in the field of interest
Zheng XQ, Ding CY, Zou YX, Zhu HY, Wang L, Fan L, Xu W, Li JY. [Roles of PET/CT in Predicting the Prognosis of Diffuse Large B Cell Lymphoma Patients Treated with Chimeric Antigen Receptor T Cell Therapy]. Zhongguo Shi Yan Xue Ye Xue Za Zhi. 2020 Aug;28(4):1189-1196. Chinese. doi: 10.19746/j.cnki.issn.1009-2137.2020.04.018.	Original article not in the field of interest
Kaminek M, Meluzin J, Panovský R, Metelkova I, Budikova M, Richter M. Long-term results of intracoronary bone marrow cell transplantation: the potential of gated sestamibi SPECT/FDG PET imaging to select patients with maximum benefit from cell therapy. Clin Nucl Med. 2010 Oct;35(10):780-7. doi: 10.1097/RLU.0b013e3181e4d9c5.	Original article not in the field of interest
Agelopoulos K, Richter GH, Schmidt E, Dirksen U, von Heyking K, Moser B, Klein HU, Kontny U, Dugas M, Poos K, Korsching E, Buch T, Weckesser M, Schulze I, Besoke R, Witten A, Stoll M, Köhler G, Hartmann W, Wardelmann E, Rossig C, Baumhoer D, Jürgens H, Burdach S, Berdel WE, Müller-Tidow C. Deep Sequencing in Conjunction with Expression and Functional Analyses Reveals Activation of FGFR1 in Ewing Sarcoma. Clin Cancer Res. 2015 Nov 1;21(21):4935-46. doi: 10.1158/1078-0432.CCR-14-2744.	Original article not in the field of interest
Rioja Zuazu J, Rodríguez M, Rincón Mayans A, Sansi AS, Zudaire Bergera JJ, Martínez-Monge R, Richter JA, Berián Polo JM. Valor de la PET en la recurrencia del cáncer de próstata con PSA < 5 ng/ml [Usefulness of PET scans in diagnosing recurrent prostate cancer. Prostate with PSA level < 5 ng/ml]. Actas Urol Esp. 2009 Sep;33(8):844-52. Spanish. doi: 10.1016/s0210-4806(09)72870-0.	Original article not in the field of interest
Martí-Climent J, Peñuelas I, Calvo R, Giménez M, Gámez C, Richter J. Utilización de un ciclotrón para la producción de radionucleidos emisores de positrones [Use of a cyclotron in the production of positron emitting radionuclides]. Rev Esp Med Nucl. 1999 Aug;18(4):261-7	Original article not in the field of interest

Meller B, Sommer K, Gerl J, von Hof K, Surowiec A, Richter E, Wollenberg B, Baehre M. High energy probe for detecting lymph node metastases with 18F-FDG in patients with head and neck cancer. Nuklearmedizin. 2006;45(4):153-9.	Original article not in the field of interest
Richter JA, Torre W, García MJ, Aramendia JM, Giménez M, Brugarolas A. PET-FDG in the Preoperative Evaluation of Non Small Cell Lung Cancer (NSCLC) Staging. A Comparison with CT Scanning and MRI. Clin Positron Imaging. 1998 Sep;1(4):247. doi: 10.1016/s1095-0397(98)00037-5.	Original article not in the field of interest
Tabata R, Iwama H, Tabata C, Yasumizu R, Kojima M. CD5- and CD23-positive splenic diffuse large B-cell lymphoma with very low CD20 expression. J Clin Exp Hematop. 2014;54(2):155-61. doi: 10.3960/jslrt.54.155.	Original article not in the field of interest
Beylergil V, Simmons MZ, Ulaner G, Jurcic J, Hibshoosh H, Carrasquillo JA. FDG PET/CT findings in a rare case of giant fibrovascular polyp of the esophagus harboring atypical lipomatous tumor/well-differentiated liposarcoma. Clin Nucl Med. 2014 Mar;39(3):288-91. doi: 10.1097/RLU.0000000000000358.	Case report not in the field of interest
Richter GT, Mennemeier M, Bartel T, Chelette KC, Kimbrell T, Triggs W, Dornhoffer JL. Repetitive transcranial magnetic stimulation for tinnitus: a case study. Laryngoscope. 2006 Oct;116(10):1867-72. doi: 10.1097/01.mlg.0000234936.82619.69.	Case report not in the field of interest
Khashab T, Sehgal L, Medeiros LJ, Samaniego F. Spontaneous regression of interdigitating dendritic sarcoma in a patient with concurrent small lymphocytic lymphoma. BMJ Case Rep. 2015 Jun 12;2015:bcr2014209014. doi: 10.1136/bcr-2014-209014.	Case report not in the field of interest
Richter WS. Imaging biomarkers as surrogate endpoints for drug development. Eur J Nucl Med Mol Imaging. 2006 Jul;33 Suppl 1:6-10. doi: 10.1007/s00259-006-0129-z.	Editorial not in the field of interest
Schuler MK, Richter S, Platzek I, Beuthien-Baumann B, Wieczorek K, Hamann C, Mohm J, Ehninger G. Trabectedin in the neoadjuvant treatment of high-grade pleomorphic sarcoma: report of a rare case and literature review. Case Rep Oncol Med. 2013;2013:320797. doi: 10.1155/2013/320797.	Case report not in the field of interest
Mahmood S, Martínez de Llano SR, Sajid S. False positive FDG-PET extensive diffuse abdominal tracer activity in a patient with CLL on whole-body 18FDG-PET/CT indicative of Ritcher's transformation. Nuklearmedizin. 2009;48(6):N69-70.	Case report in the field of interest

Torre W, Garcia-Velloso MJ, Galbis J, Fernandez O, Richter J. FDG-PET detection of primary lung cancer in a patient with an isolated cerebral metastasis. J Cardiovasc Surg (Torino). 2000 Jun;41(3):503-5.	Case report not in the field of interest
Gorospe Sarasúa L, Jaureguízar-Oriol A, Almonacid-Sánchez C, Rioja-Martín ME. Richter Syndrome With Extensive Isolated Pleural Extranodal Involvement: The Importance of PET/CT Imaging. Arch Bronconeumol. 2017 Nov;53(11):644-646. English, Spanish. doi: 10.1016/j.arbres.2017.03.006.	Case report in the field of interest
Betts AM, Banks KP, Solberg AO. Unsuspected perforated Richter hernia in the inguinal canal detected by F-18 FDG PET/CT. Clin Nucl Med. 2011 Dec;36(12):1118-9. doi: 10.1097/RLU.0b013e3182335ddf.	Case report in the field of interest
Gajendra S, Gogia A, Tanwar P, Sahoo MK, Bhethanabhotla S, Durgapal P, Gupta R. Synchronous metastatic pulmonary adenocarcinoma with small cell lymphoma. Leuk Lymphoma. 2014 Jul;55(7):1678-80. doi: 10.3109/10428194.2013.850166.	Case report not in the field of interest
Banzo J, Ubieto MA, Giraldo P, Yus C, Santapau A, Parra A. Hallazgo accidental de un adenoma túbulo-velloso de recto mediante (18)F-FDG PET en un paciente diagnosticado de linfoma linfocítico [Accidental finding of a tubulovellous adenoma of rectum by 18F-FDG PET in a male patient diagnosed of lymphocytic lymphoma]. Rev Esp Med Nucl. 2011 Jul-Aug;30(4):256-7. Spanish. doi: 10.1016/j.remn.2010.10.014.	Case report not in the field of interest
Bernardi S, De Nardis L, Macci E, Fiorelli M, Francia A. Multi-domain cognitive impairment disclosed before the diagnosis of chronic lymphocytic leukaemia: an autoimmune/paraneoplastic disorder? Neurol Sci. 2013 May;34(5):801-3. doi: 10.1007/s10072-012-1141-7.	Case report not in the field of interest
